# Experimental Evolution Reveals Genome-Wide Spectrum and Dynamics of Mutations in the Rice Blast Fungus, *Magnaporthe oryzae*

**DOI:** 10.1371/journal.pone.0065416

**Published:** 2013-05-31

**Authors:** Junhyun Jeon, Jaeyoung Choi, Gir-Won Lee, Ralph A. Dean, Yong-Hwan Lee

**Affiliations:** 1 Department of Agricultural Biotechnology, College of Agriculture and Life Science, Seoul National University, Seoul, Korea; 2 Fungal Bioinformatics Laboratory, Seoul National University, Seoul, Korea; 3 Department of Bioinformatics and Life Science, Soongsil University, Seoul, Korea; 4 Functional Genomics Program, North Carolina State University, Raleigh, North Carolina, United States of America; 5 Center for Integrated Fungal Research, Department of Plant Pathology, North Carolina State University, Raleigh, North Carolina, United States of America; 6 Center for Fungal Pathogenesis, Seoul National University, Seoul, Korea; 7 Center for Fungal Genetic Resources, Seoul National University, Seoul, Korea; Yonsei University, Korea, Republic of

## Abstract

Knowledge on mutation processes is central to interpreting genetic analysis data as well as understanding the underlying nature of almost all evolutionary phenomena. However, studies on genome-wide mutational spectrum and dynamics in fungal pathogens are scarce, hindering our understanding of their evolution and biology. Here, we explored changes in the phenotypes and genome sequences of the rice blast fungus *Magnaporthe oryzae* during the forced *in vitro* evolution by weekly transfer of cultures on artificial media. Through combination of experimental evolution with high throughput sequencing technology, we found that mutations accumulate rapidly prior to visible phenotypic changes and that both genetic drift and selection seem to contribute to shaping mutational landscape, suggesting the buffering capacity of fungal genome against mutations. Inference of mutational effects on phenotypes through the use of T-DNA insertion mutants suggested that at least some of the DNA sequence mutations are likely associated with the observed phenotypic changes. Furthermore, our data suggest oxidative damages and UV as major sources of mutation during subcultures. Taken together, our work revealed important properties of original source of variation in the genome of the rice blast fungus. We believe that these results provide not only insights into stability of pathogenicity and genome evolution in plant pathogenic fungi but also a model in which evolution of fungal pathogens *in natura* can be comparatively investigated.

## Introduction

Propagation of fungi by subculture is routine in many laboratories. One pervasive idea that is rarely tested in the studies of microbial organisms such as fungi is that this practice without selection under laboratory settings maintains clonality. Studies such as Kohn *et al*. [1] and Park *et al.* [2] supported this view, although their conclusions are based on the use of handfuls of markers. This assumption is of particular importance in genetic analysis where functions of gene are inferred from observed phenotypes of mutants having the target gene deleted or mutated. For this inference to hold, the phenotypes should be attributed solely to the mutation introduced. If other mutations occur spontaneously during subculture or preparation of stock cultures, then the new mutations may induce secondary phenotypes or affect phenotypes of interest through genetic interactions with the solicited mutation. However, the rate at which such mutations occur and the molecular spectrum of their effects are largely unknown. Furthermore, selection that may be imposed by growing the fungus in the artificial media has not been appreciated at the molecular level to date.

*Magnaporthe oryzae* is the causal agent of rice blast disease that is one of the most devastating diseases in rice plants, and is a model plant pathogenic fungus with a completely sequenced ∼41 Mb nuclear genome [3]. The fungus usually disseminates via asexual spore, called conidia that on a leaf surface, produce a germ tube in the presence of water. The germ tube tip develops into a specialized infection structure, the appressorium, in response to environmental cues such as surface hydrophobicity [4]. Using turgor pressure within the appressorium, the fungus mechanically penetrates into host plant and grows inside it, leading to formation of disease lesions where the fungus produces conidia for secondary infection. This infection kills host tissues, causing yield losses or even death of the plant. It was reported that during single rice growing season, fungal infection cycles 8 to 11 times under the field condition [5]. Although conidia are considered a major source of inoculum, vegetative hyphae can also form appressorium and cause disease when inoculated onto host plants as a recent study demonstrated [6].

The deployment of resistant cultivars is a primary means of controlling the rice blast disease. However, new fungal strains rapidly overcome resistance, leading to disease epidemics in the field [7]. Insertion of a transposon into an avirulence gene was demonstrated to be one cause of resistance breakdown [8], yet this doesn't seem to be the prevailing mechanism. A number of other explanations are possible to explain resistance resistance breakdown. For example, 1. Mutation rate accelerates when selection pressure is imposed by introduction of resistance cultivars; 2. Population of *M. oryzae* in the field is genetically diverse and introduction of resistance cultivars simply confers selective advantage on a particular subpopulation that has mutations in genes including ones conferring avirulence. These represent two different mechanisms, but they are not mutually exclusive. Examining nucleotide sequences of populations that have survived many cycles of natural selection imposed by interaction with the host plant may provide clues to the underlying mechanism. Subsequently, detecting the signature of selection from such datasets requires the knowledge on the properties of original source of variation: spectrum and dynamics of DNA mutations.

A method that enables examination of the spontaneous mutations in an organism is the mutation accumulation (MA) experiment [9], in which mutations are allowed to drift to fixation. During the MA experiment, genetic drift that is created by population bottleneck prevents the spontaneous mutations from being purged by natural selection, even if those mutations are slightly deleterious [10]. The MA experiment has been successfully used to investigate the rate and spectrum of spontaneous mutations in model organisms such as *Echerichia coli*, *Saccharomyces cerevisiae*, *Caenorhabditis elegans*, and *Arabidopsis thaliana* [11], [12], [13], [14], [15], [16]. However, we propose that the MA experiment cannot be applied to filamentous fungi due to the cellular and genetic properties specific to the filamentous fungi. Unlike single-celled fungi such as yeast, individual cells in filamentous fungi do not exist in isolation but are physically connected to one another, forming a network structure, called hyphae. As a result, individual nuclei are not independent from one another. In combination with parasexualism [17], [18], this network structure provides the means for filamentous fungi to combine mutations from different nuclei, preventing or attenuating the effect of clonal interference [19], [20]. In addition, many filamentous fungi lack a sexual cycle in the field, rendering it difficult to define a generation in these multi-cellular eukaryotes.

Here we modified the MA experiment to serially passage a mass of fungal tissue on artificial media and used it to characterize the experimental evolution of *M. oryzae*. Phenotypic changes observed in the derived strains suggest that the deleterious effects of mutations seem to be effectively buffered by the fungal genome. Analysis of whole genome sequencing data show that mutations in the fungus accumulate more rapidly than previously thought and selection as well as genetic drift are acting to shape the mutational spectrum during subculture of the fungus on artificial media. The resulting mutational spectrum suggests that the main sources of mutations during subculture under the laboratory conditions are probably oxidative damages and UV, suggesting higher mutation rate in field environments. To our knowledge, this is the first characterization of the experimental evolution of filamentous fungi at single-nucleotide resolution. We believe this work would not only provide basic information in better understanding and predicting evolutionary path of *M. oryzae* but also aid in interpretation of genetic analysis for the fungus.

## Results

### Experimental evolution via serial passage of fungal cultures

Previous studies using the MA experiment in model organisms subjected the population to an extreme degree of bottleneck. For example, only a single seed for the plant or a single cell for the yeast was used to establish the next generation. This enables any mutations within the genome of the selected individual to be fixed quickly. New mutations accumulate in addition to the fixed mutations. Such extreme bottleneck is useful in estimating mutation parameters such as mutation rate. However, this extreme bottleneck is unlikely in natural and laboratory settings where filamentous fungi are studied. In many laboratories, subculture of the fungus is a routine practice carried out by transferring a portion of existing culture, leading inevitably to genetic drift of some extent.

**Figure 1:**
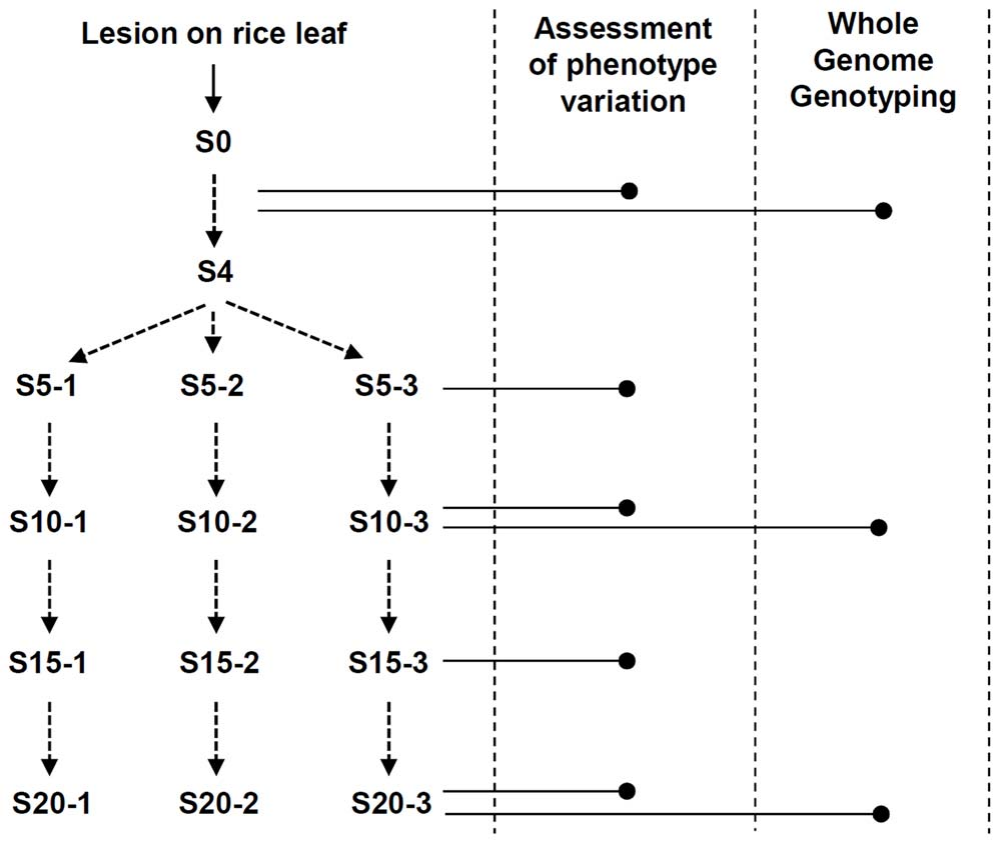
Schematic diagram of serial passage experiment in *M. oryzae*. S0 is the culture of the fungus isolated from a disease lesion on a rice leaf. A patch of culture was transferred and spread out every week to the new oatmeal agar plates comprising the next subculture. Subculturing continued until it reaches S20. From S4, three independent cultures were maintained. Subcultures subjected to phenotype assessment and genotyping were indicated by each horizontal line with dot at its right end.

In order to understand what mutations occur at what rate and how these mutations affects the traits of the fungal pathogen, *M. oryzae*, we modified the MA experiment by serially transferring a portion of cultures instead of single conidia (asexual spore with three cells) on oatmeal agar plates. The laboratory strain 70-15 was used due to the availability of genome sequence information. The serial passage experiment was done as described in Figure 1. The initial culture was derived from a disease lesion caused by the fungus on a rice leave to ensure the full virulence and was designated S0. The S0 was subcultured on oatmeal agar plate, forming S1. The subculturing continued up to S20. Starting from S4, the fungus was subcultured in three independent lineages (named lineage 1, lineage 2, and lineage 3). Having multiple lineages is expected to give more information on mutation processes of this serial passage experiment than using a single lineage. All the derived strains were stored as permanent stock cultures in −70°C for later uses. After the derived strains were established, phenotypic changes were first monitored during the experimental evolution of the fungus.

### Colony morphology and growth rate of the derived strains

**Figure 2:**
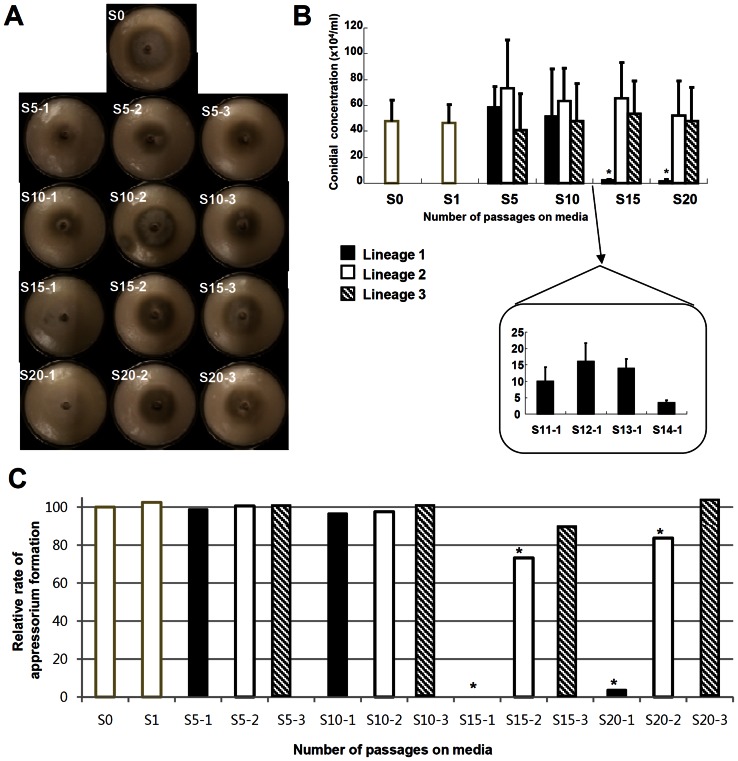
Phenotypic changes of the derived strains. (A) Colony morphologies of the derived strains on oatmeal agar plates. S0 and every fifth derived strains are shown. (B) Asexual reproduction of the derived strains. Five replicates were used to quantify asexual reproduction. Asexual spore production of S11-1 to S14-1 was shown below (inset). (C) Relative appressorium formation rate of the derived strains to that of S0. Taking the percentage of appressorium formation of S0 as 100, the relative rate of the derived strains were calculated and plotted. Asterisk indicates statistically significant differences, compared to S0 (Tukey test with P<0.05).

Since it is laborious and time-consuming to check the phenotypic changes of all the derived strain, the phenotypes of S0 were compared with every fifth derived strains (S5, S10, S15, and S20) (Figure 1). Colony morphology was checked first. Because colony morphology is subject to small changes in the environment, the norm of reaction for colony morphology of wild-type was explored carefully. Based on the wild-type norm of reaction, seemingly aberrant morphology of the derived strains was repeatedly checked for reproducibility. We confirmed that in the lineage 1, aerial hyphae and mycelia melanin decreased, and that in the lineage 2, mycelia melanin was excessive (Figure 2A). Colony morphology of the lineage 3 didn't change, compared to that of wild-type. The observed changes in the lineages 1 and 2 started to appear at S15. However, the growth rate didn't show dramatic differences among the derived strains (Figure S1).

### Production of asexual spores, germination and appressorium formation in the derived strains

Examination of asexual spore production revealed that the lineage 1 of S15 and S20 barely produced conidia (Figure 2B). To find out when this impairment began, conidiation of the lineage 1 in S11 to S14 was quantified. We found that S11-1 already showed significant reduction in conidiation (25% of S0) and conidiation of S14-1 was comparable to that of S15-1.

Germination from conidia was normal for every derived strain (Figure S2). Appressorium formation started to be defective from S_15_. Especially in lineage 1, almost no conidia developed appressoria (Figure 2C and Figure S2).

### Virulence of the derived strains

**Figure 3:**
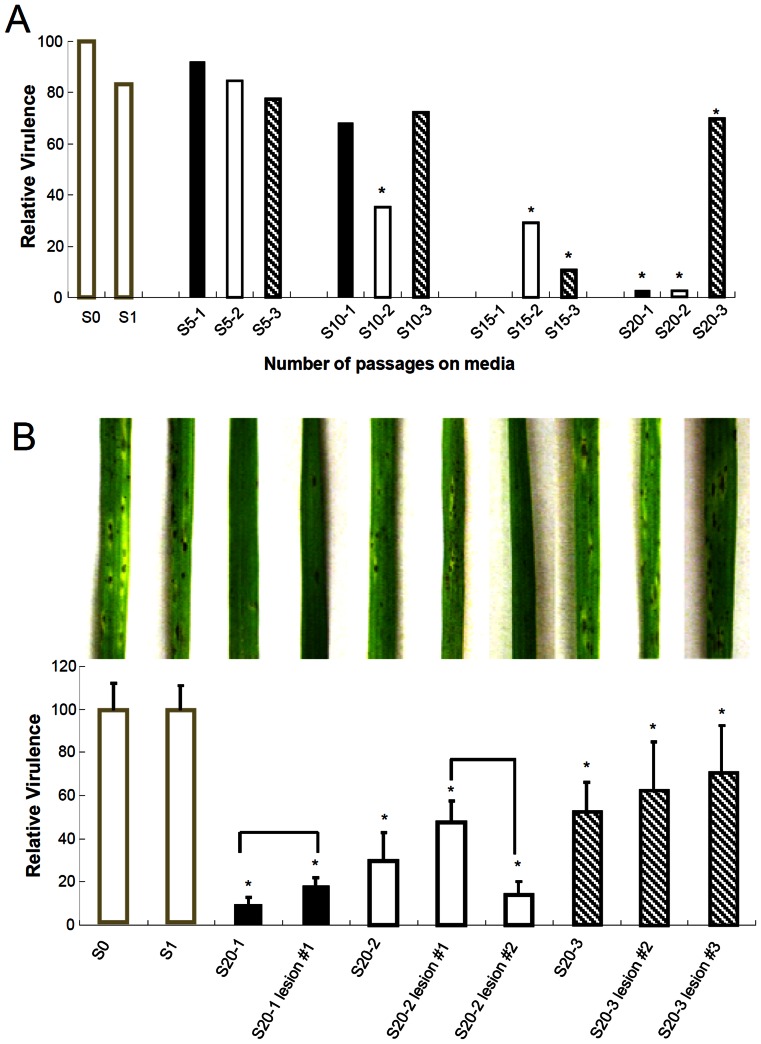
Virulence of the derived strains. Relative virulence of the derived strains (A) and lesion isolates from the derived strains (B) to that of S0 is shown. Taking virulence of S0 as 100, the relative virulence of the derived strains and their lesion isolates were calculated and plotted. Asterisks indicate statistically significant difference between S_0_ and the derived strains and their lesion isolates (t test with P<0.05). Statistical difference within each group consisting of the derived strain and its lesion isolates was indicated by vertical brackets (Tukey test with P<0.05).

Unlike colony morphology, conidiation, and appressorium formation, the reduction in virulence was first observed in S10-2 (Figure 3A and Figure S3). The defect in virulence was exacerbated in S15 and S20, regardless of the lineages. The derived strains in S15 and S20 didn't cause as many lesions as S0 and the sizes of lesions were smaller than those of S0 (Figure 3A and Figure S3). It is noteworthy that the virulence of the lineage 3 was greatly reduced in S15 but significantly restored in S20, though it was still less virulent than S0. Except the lineage 1 whose appressorium formation rate is less than 10%, the other derived strains showed appressorium formation rate of over 70%. This suggests that the reduced virulence cannot be attributed solely to the defect in appressorium formation. It is conceivable that mutations that occurred during the serial passage experiment may be involved in appressorium-mediated penetration of cuticle layer or invasive growth inside host cells.

The reduced lesions observed on leaves inoculated with the derived strains pose an interesting question about the relationship between virulence and genetic heterogeneity of the population. Our serial passage experiment would allow unfixed mutations to remain in the population. Assuming the presence of unfixed mutations that may affect the virulence, are the lesions caused by subpopulation that doesn't have those mutations? If that's the case, one prediction is that the fungus isolated from the lesions would be more virulent than the strain it is derived. To test this, we first isolated the fungus separately from the lesions caused by S20-1, S20-2, and S20-3. The lesions caused by S0 and S1 were included as a control. We tried at least three lesions for each derived strain but obtained only one for S20-1 or two isolates for S20-2 and S20-3, suggesting that some of lesions caused by the derived strains are not viable. Spores were harvested from these lesion isolates and used for spray-inoculation onto rice plants. Interestingly, the five lesion isolates seemed to be more virulent than the derived strains themselves, although the differences were not statistically significant (Figure 3B). This result supports our prediction about genetic heterogeneity of the population.

### Genome-sequencing of the derived strains

In an attempt to investigate the mutations that have occurred during the MA experiment, DNA fingerprinting was carried out for the derived strains and their lesion isolates using MGR586 [21] as a probe. Our DNA fingerprinting analysis, however, revealed little differences among strains tested (Figure S4). This suggests that the derived strains are really ‘derived’ from S0 and resolution of fingerprinting approach may be too low to detect mutations.

To catalogue the mutations within the genome of the derived strains at single nucleotide resolution, we employed next generation sequencing technology for sequencing of S0 (1 strain), S10 (3 strains), and S20 (3 strains). Genomic DNAs of mycelia tissues were extracted and sequenced, yielding sequencing reads that correspond to about 30 to 100× coverage (Table S1). Large differences in amount of sequencing reads among the derived strains were mainly due to the use of different version of sequencing platforms: Illumina GAIIx for S0 and S20-1, and HiSeq system for the rest. To deal with possible biases resulting from vastly uneven sequencing reads, we randomly selected the sequencing reads to make raw read depth equal (∼40× except S_0_) (Table S2). Our sequencing data covered at least 96% of nucleotide sites within the fungal genome (Table S3). Since it is not known how identical S0 is to the strain used for establishing reference genome sequences, we aligned the resulting S0 sequences to the references genome and subtracted the existing mutations, making a corrected reference genome. Sequencing reads from the derived strains were aligned to this corrected reference genome.

To call mutations, only the sites covered with at least four but less than 100 sequencing reads were considered. This allowed us to call mutation with high confidence for on average 96.3% of nucleotide sites in the genome (Table S4). By the nature of the experiment, we expected to see different types of mutations in terms of fixation: Some of the mutations would be fixed in the population and the others would not yet fixed or in the course of fixation. We reasoned that low frequency mutations would complicate our analysis because they are likely to be discarded during genetic drift and are unlikely to have major effects on phenotypic changes of the derived strains. Furthermore, calls of low frequency mutation can be dubious due to the possible sequencing error. Therefore, we decided to restrict our analysis to the mutations having frequency of ≥0.5.

To validate our high throughput sequencing data, we randomly selected 18 predicted mutations for Sanger sequencing (Table S5). One of the loci was not amplified in the PCR. For the remaining 17 loci that were successfully amplified, we sequenced the PCR products and compared the resulting sequences with the corrected references at the corresponding loci by BLAST search. From fifteen loci, we confirmed the variations predicted by high throughput sequencing method. For the other two loci, two independent methods didn't agree. One of the two loci was unfixed mutation, providing the possibility that PCR was somehow biased toward the one base against the other. Our validation experiment suggests that our dataset is reliable enough to carry out further analysis.

### Mutations that occurred during serial passage experiment among the derived strains

**Figure 4:**
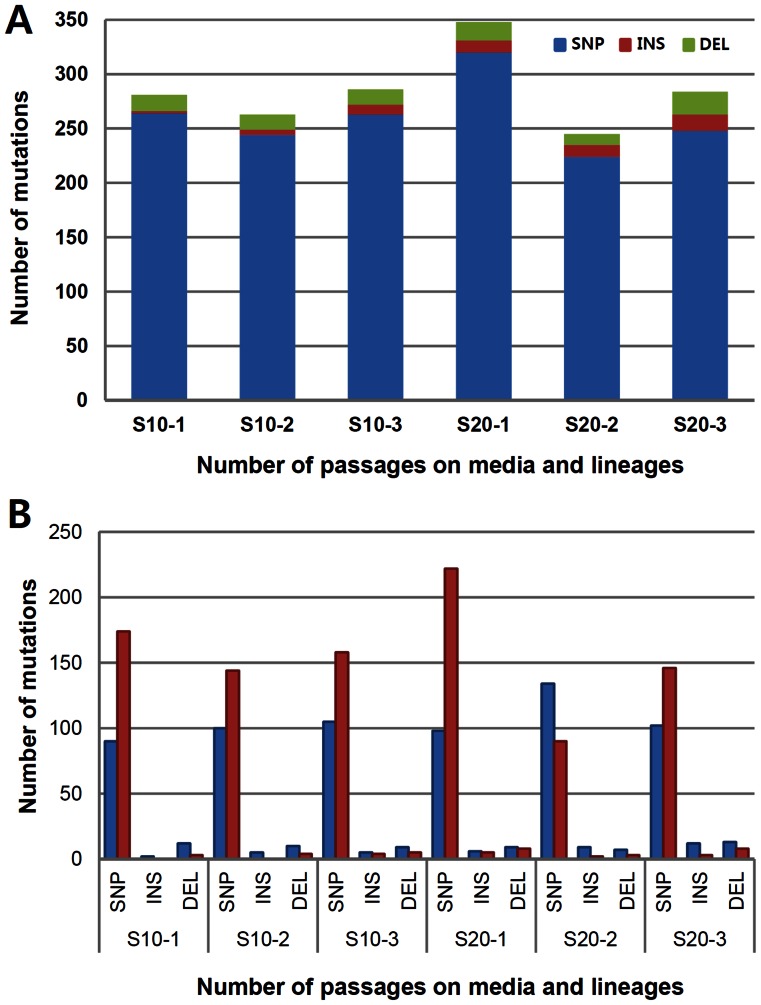
The number and types of mutations in the derived strains. (A) Number of mutations in the derived strains. Blue, red, and green indicate single nucleotide polymorphism (SNP), insertion (INS), and deletion (DEL), respectively. (B) Types of mutations in the derived strains. Blue and red bars represent fixed and unfixed mutations according to sequencing read over the nucleotide site: mutation call frequency of 1 is designated as ‘fixed’ and the mutation call frequency between 0.5 and 1 as ‘unfixed’.

By applying the read depth and frequency criteria, we were able to identify varying number of mutations from the derived strains (Figure 4A). Overall, the number of mutations was between 200 and 350 per strain. The smallest number was found in S20-2 and the largest was in S20-1. Interestingly, the number of mutations didn't seem to be proportional to the number of subcultures. Regardless of lineages, the number of mutations in the derived strain reached about 250 at S10 and didn't increase significantly in S20 except in S20-1. Among all the mutations detected, more than 90% of mutations were single nucleotide polymorphisms (SNP) and, insertions and deletions were less than 10% of overall mutations. Around 30 to 60% of mutations were fixed in the population, depending on the strains (Figure 4B). This mixture of fixed and unfixed mutation is what we expected to see and reflects the experimental design involving population of cells rather than a single cell. Most of insertions and deletions were one or two bases long (Figure S5).

### Mutational bias among chromosomes

**Figure 5:**
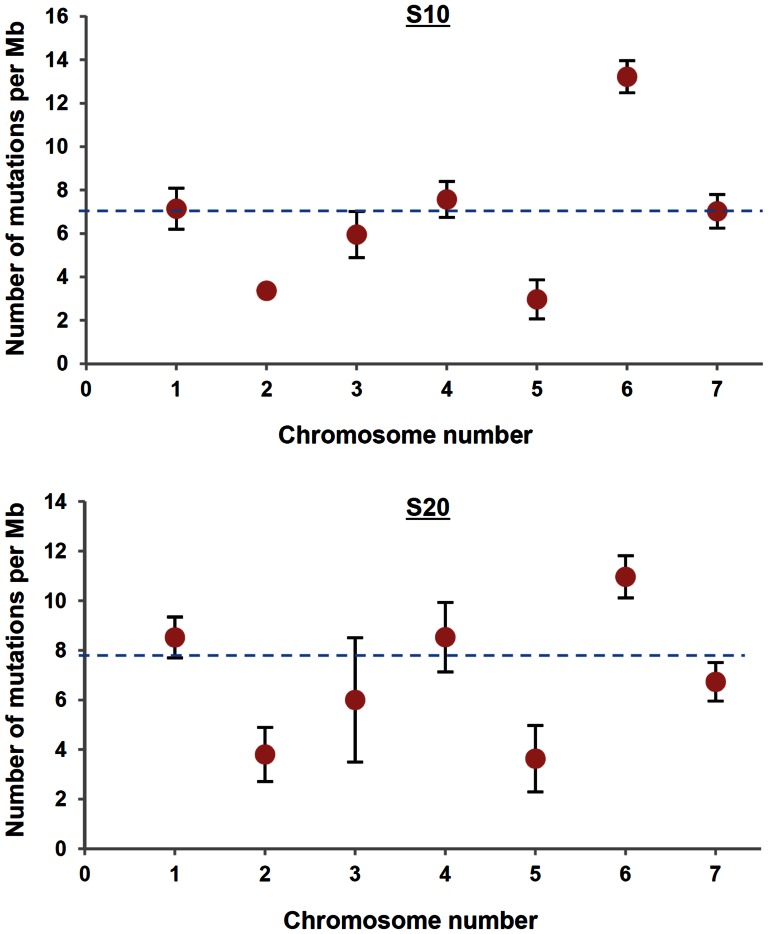
Mutational bias among chromosomes of the derived strains S10 (upper panel) and S20 (lower panel). X-axis indicates seven chromosomes and y-axis indicates the number of mutations per Mb. The dotted lines represent the expected number of mutation.

Given the number of mutations, we asked if mutations were evenly distributed among chromosomes by comparing the number of observed mutations with the expected number among chromosomes (Figures 5 and Figure S6). The expected number of mutations in each chromosome was calculated based on the length of corresponding chromosome. We found that there was a significant bias in the distribution of mutations among chromosomes (χ^2^ test, 6 df, P = 2.71×10^−17^ for S10 and P = 1.27×10^−16^ for S20). Chromosomes 1, 4, and 6 tended to have more mutations than expected, whereas chromosomes 2 and 5 tended to have less than expected. Large standard deviation in S_20_ is due to the higher number of mutations in S20-1 than in other lineages. It is not clear what caused this bias among chromosomes.

### Dynamics of mutations among and between lineages

**Figure 6:**
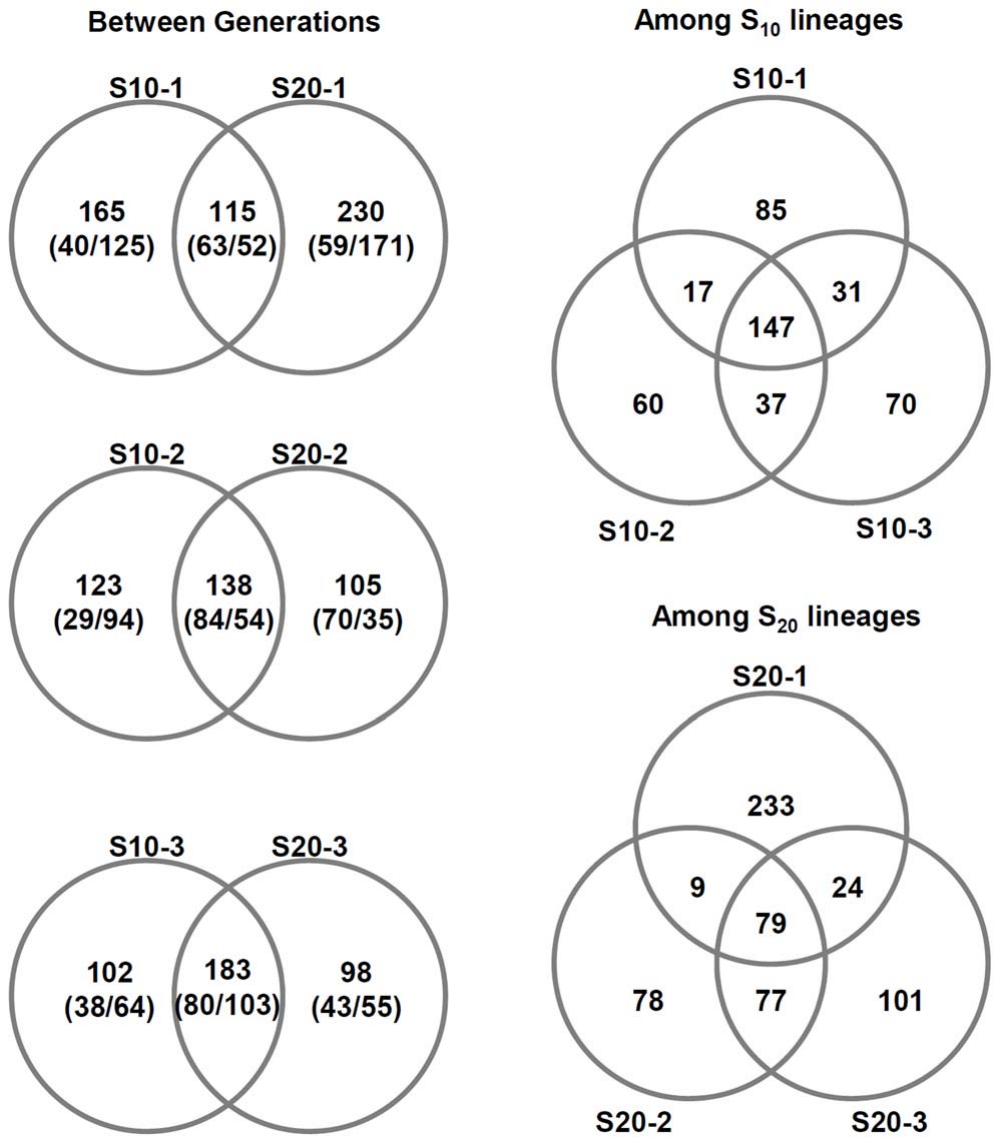
Mutations that overlap among lineages or between generation. (#1/#2): #1, number of fixed mutations; #2, number of unfixed mutations.

To explore the dynamics of mutations, we examined how many mutations overlap between S10 and S20 or among lineages (Figure 6). Over 50% of mutations were shared between S10 and S20 except the case between S10-1 and S20-1. As expected, many mutations that were present in S10 but lost in S20 were not fixed ones, showing that even the medium frequency mutations have high probability of being lost during subcultures. Notably, new mutations occurred in S20, while some of the mutations that existed in S10 were being lost (left column of Figure 6). Since the number of mutations appeared to increase rapidly before S10 and stabilize in S20, we speculate that mutation rate is initially high during the course of subcultures and later becomes low to reach equilibrium where the rate of occurrence and extinction become equal.

In our experiment, we kept three independent lineages. Comparison of the mutations in three lineages showed that a significant portion of mutations (∼30 to 70%) were conserved among them (right column of Figure 6). This suggests two possibilities that are not mutually exclusive. First, the genome of *M. oryzae* has mutational hotspots that mutate more frequently than other sites. Alternatively, selection is acting on the genome to retain mutations at a particular set of sites. As a way to test whether selection was affecting mutations, we examined the distribution of mutations in coding sequences and non-coding sequences. Our expectation under no selection was that the mutations would be even distributed between coding and non-coding sequences in proportion to their length. We calculated the expected number of mutations in coding and non-coding sequences and compared it with the observed number of mutations in each region (Table S6). The result showed strong bias toward the non-coding sequences (χ^2^ test, 3 df, P<1×10^−10^ for all cases), indicating that selection might have driven out the mutations in the coding sequences. It is uncertain whether selection is due to oatmeal agar plate or within-population competition. Although the selection might be acting on, we cannot rule out the possibility that there are mutational hotspots especially in the non-coding part of genome of *M. oryzae*. However, we don't know whether, under different experimental settings, the observed mutations can be replicated by repeated subcultures.

### Transition vs. Transversion

**Figure 7:**
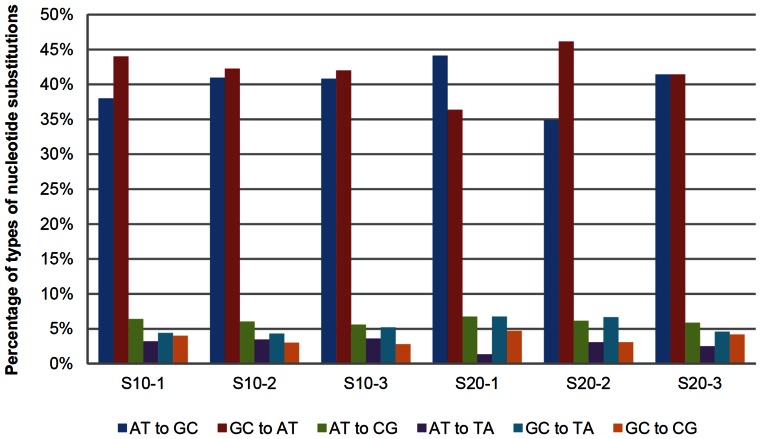
Percentage of types of nucleotide substitutions in the derived strains.

We analyzed the mutational spectrum in the derived strains by considering the six base-substitution types. We found that, in all derived strains, the rate of transition (Ts) base-substitution are nearly four to five times as many as transversion (Tv) base substitutions, leading to the Ts/Tv ratio between 4 and 5 (Figure 7 and Table S7), which is much greater than the random expectation of 0.5. This indicates the overabundance of transitions, compared to transversions. In transitions, G:C → A:T and A:T → G:C occurred at similar rate. Although we don't know the particular sources of DNA damage or error leading to A:T → G:C, it is known that spontaneous deamination of cytosine or 5-methylcytosine is the major source of G:C → A:T transitions [22], [23]. However, DNA methylation is unlikely to be a major cause of this because methylated cytosine accounts for only 0.2% of total cytosines (unpublished data). We propose that UV-induced mutation and/or oxidative damage that can cause G:C → A:T transitions [24] played a major role in mutagenesis of the derived strains.

### Contribution of mutations to phenotypic changes

To relate the mutations to the phenotypic changes observed in the derived strains, we took advantage of information from the literatures and T-DNA insertions mutants we have in the lab. We found that two genes among the ones having mutations in the derived strains were reported in the literatures. Those were *THL1* and MgCdc42 [25], [26]. Mutations in two genes were found in all the derived strains examined. The mutations were either in the promoter region (*THL1*) or intron (*MgCdc42*), thus rendering it difficult to make direct connection between mutations and phenotypes, although we cannot exclude the possibility that the mutations can interfere with transcription or splicing.

We therefore turned to T-DNA resources. Over 20,000 strains of T-DNA insertion mutants and their phenotypes are archived in Center for Fungal Genetics Resources (http://knrrb.knrrc.or.kr/index.jsp?rrb=cfgr) and ATMT database (http://atmt.snu.ac.kr) [27]. To make use of these resources, we extracted the annotated genes that had mutations in exon sequences of the derived strains and were tagged by T-DNA at the same time (Table S8). A total of seven annotated genes were found to meet these criteria. Most of mutations were amino-acid changing i.e. non-synonymous mutations. However, we also found a synonymous mutation, a frame-shift mutation by insertion, and a codon deletion mutation by nucleotides deletion. The derived strain that have the most mutations was S20-1, which is consistent with the fact that S20-1 displayed more phenotypic defects than any other strains. T-DNA insertions in genes having mutations caused defects in growth, appressorium formation, and pathogenicity. Since T-DNA insertions are mostly in promoter regions of the genes, they would probably affect the transcription of downstream genes. So T-DNA insertions would have different effects at the molecular level from mutations we found in the derived strains. However, phenotypic consequences of those effects are likely to be similar to each other. We thus conclude that these mutations would have likely contributed to the phenotypic changes observed in the derived strains.

## Discussion

For genetic studies of fungi, subcultures on artificial media under laboratory conditions are routine practice. The idea or assumption that underlies subculture practice is that fungal culture regenerated from clonally related isogenic founders is stable and thus invariant at both phenotypic and genotypic level. However, this idea has been rarely tested and no whole-genome scale investigation has been undertaken. In this study, we monitored the changes in phenotypes and genome sequences that occurred during repeated *in-vitro* subcultures of *M. oryzae* on oatmeal agar media.

We expected that mutations in DNA sequences involved in conidation, appressorium formation, and virulence would be nearly neutral because the fungus is propagated by human intervention and is away from its host plants. Consistent with our expectation, we observed the defects in aforementioned traits. However, phenotypes were quite stable, since little changes were detected until S10. This observation is consistent with the previous report [2], though they used single-spore as an inoculum instead of a patch of fungal culture. In our high throughput sequencing data, 200–350 mutations per the derived strain was detected. The number of mutations seemed to increase rapidly up to S10 and reach equilibrium somewhere between S10 and S20. Although it is difficult to interpolate with a few data points, it is tempting to speculate that accumulation of mutations and their phenotypic changes display opposing pattern during the course of experimental evolution. This discordance may reflect the capacity of fungal genome to buffer the mutations. That is, certain number of mutations is somehow tolerated and more mutations than a critical number are deleterious to the fungus, probably through genetic interactions.

After S10, all three lineages showed varying degree of defects in different traits and these defects were exacerbated as later generations go, suggesting the effect of genetic drift and accumulation of deleterious mutations during subcultures. Indeed, our sequencing data showed the effect of genetic drift: distinct mutations in different lineages. However, a significant portion of mutations were shared among lineages, suggesting the action of selection. We reasoned that if selection is acting on the genome of the derived strains, then we would see the bias in distribution of mutations between coding and non-coding sequences. Comparison of number of mutations in coding and non-coding sequences revealed strong mutational bias toward the non-coding sequences, implying the presence of selection against the mutations in coding sequences. These results indicate that mutational landscape of the fungal genome under subculture scheme is shaped and maintained by both genetic drift and selection.

One question that arises from our data is what would happen if S10 or S20 cultures were to become founding population and were sub-cultured for a long time. Will they accumulate new mutations rapidly up to next 10 generations and reach equilibrium again as they did before? Or will they stay in equilibrium as they appear now? One clue to the question is that there were already many mutations (over 500 mutations) in S0, compared to the reference genome sequences of 70-15. Although we don't know the exact history of S0 before the experiment was undertaken, it is obvious that the fungus went through repeated subcultures since the genome sequencing of original strain. This suggests that mutations randomly occur in the genome but selection from growing the fungus on artificial media can largely determine the set of mutations that are retained in the genome.

Our virulence test with the lesion isolates of the derived strains suggested the genetic heterogeneity of population in the fungal culture. It has been recognized that the fungus can cause different types of lesions on the single leaf of host plant [28]. Different lesion types can be attributed either to differences in micro-environment of plant surface where the fungal spores are placed or to difference in genotypes of fungal spores inoculated. The relative contribution of the two factors to the lesion formation is unclear, but our data suggest that genetic heterogeneity at the population level can explain variation in disease lesions to some extent.

One of our predictions during the experiment was that we might be able to see mutations in known pathogenicity genes and/or avirulence genes if we effectively eliminate host selection by growing the fungus away from it. However, we found mutations only in an intron of *MgCdc42* and no mutations in the avirulence gene, *Avr-Pita*. This is probably because many known pathogenicity genes are also required for normal growth as well as host infection and thus supports that pathogenicity of *M. oryzae* is not a simple phenotype that can be determined by a handful of factors but a complicated biological phenomena that involves essential components of cells. No mutations in the avirulence gene may suggest either that the time given during 20 subcultures wasn't long enough for mutations to occur or that there are unknown mechanisms keeping mutation in avirulence genes in check. No avirulence genes were shown to be virulence factors in compatiable interaction of *M. oryzae*, providing an evolutionary conundrum for their maintenance in the fungal genome. Such stability of avirulence genes is the topic that deserves further works in the future.

We found that major source of mutations in *M. oryzae* during subcultures under laboratory conditions are oxidative damage and UV probably coupled with fidelity error in repair. This implies that the mutation rate in nature can be higher than that reported here, because UV radiation during our experiment was probably much lower than in natural conditions.

Taken together, we investigated the experimental evolution of the model plant pathogenic fungus, *M. oryzae* at both phenotypic and genotypic levels. We are aware of no other similar analysis of experimental evolution of filamentous fungi with single nucleotide resolution, so the generality of our observations is unknown. However, it is clear that filamentous fungi pose unique challenges in terms of life style and biology. Therefore, we believe that our endeavor would provide not only the basis for future work to be compared but also information that can aid in genetic analysis of this fungus. Our results also call attention to new opportunities for experimental evolution to explore the long-term dynamics of mutations in relation to compatible and incompatible interactions of fungi with their host plants.

## Materials and Methods

### Establishment of parental culture and serial passage culture scheme

A laboratory strain of *Magnaporthe oryzae*, 70-15 in Dr. Dean's lab was used to establish the parental culture as follows: This laboratory strain was grown on oatmeal agar plate for a week under constant fluorescent light. Spore suspension (∼5×10^4^ spores/ml) was prepared from this culture and used for spray-inoculation on a susceptible rice variety M2O2. One of the resulting susceptible lesions was excised and surface-sterilized, and transferred onto oatmeal agar plate. This plate culture was grown for 10 days and designated as parental culture (S0). A patch of S0 (∼1.5×1.5 cm) including both mycelia mass and conidia was taken and streaked on new oatmeal agar plate, and grown for a week to establish the next generation of culture (S1). This successive subculturing was repeated until it reaches S20. Starting from S_3_, subculture was done in three plates, establishing three independent lineages (S3-1, S3-2 and S3-3) to S20 (S20-1, S20-2 and S20-3). Stock culture was prepared for every culture from each generation and stored at −70°C.

### Vegetative growth, conidiation, and appressorium formation

Vegetative growth rate was measured on complete media, oatmeal agar and V8 juice agar plates on days 3 and 5 with three replicates. Ability to produce conidia was measured by counting the number of conidia from 5-day-old V8 agar plate. Conidia were collected by flooding the plate with 5 ml of sterilized distilled water. The number of conidia was counted using hemacytometer under a microscope. Conidial germination and appressorium formation were measured on plastic coverslip. Conidia were harvested from 8 to 10-day-old culture on oatmeal agar plate with sterilized distilled water. Conidial suspension of 40 µl was pipetted onto plastic coverslip following adjustment of its concentration to ∼2×10^4^ spores/ml. Drops were placed in a moistened box and incubated at 25°C. After 8 h incubation, the percentage of conidia germinating and germinated conidia forming appressoria was determined by microscopic examination of at least 100 conidia per replicate in at least three independent experiments, with three replicates per experiment.

### Pathogenicity assay

Conidia were harvested from 8 to 10-day old culture on oatmeal agar plate with sterilized distilled water. Conidial suspension was adjusted to ∼5×10^4^ spores/ml in concentration and spray-inoculated on susceptible rice seedlings (cv. M2O2) of 3 to 4 leaf stage. Inoculated plants were kept in dew chamber for 24 h and allowed to grow within growth chamber for 5 days.

### DNA isolation and manipulation

Genomic DNA for Southern hybridization and sequencing was prepared by standard method [29]. Restriction enzyme digestion, agarose gel separation, and DNA gel blotting were performed following the standard protocols [29]. Hybridization was carried out in solution containing 6×SSC, 5×Denhardt's solution, 0.5% SDS and 100 µl ml^−1^ denatured salmon sperm DNA, at 65°C. Blots were exposed to X-ray film.

### DNA sequencing and read processing

We sequenced genomes from three independent cultures derived by 10 and 20 generations of plate cultures of the reference strain 70-15, for which genome sequence information was published in 2005 [3]. Illumina (Illlumina, San Diego, CA) Genome Analyzer platform was used to obtain a depth of sequence coverage of between 30 and 100 in each culture.

In order to ensure the accuracy of further analysis, the following several steps are performed to filter the raw data (according to actual condition can be chosen). (1) Remove reads with a certain proportion of N's bases or low complexity reads (less than 10%, parameter setting at 9 bp); (2) Remove reads with a certain proportion of low quality (≤Q20) bases. (less than 40 bases); (3) Remove adapter contamination. (at least 15 bp overlap between adapter and reads, and at most 3 bp mismatches); (4) S_10_ and S_20_ lineage sequence was adjusted to the same size using random selection method. The above processes are applied to read1 and read2. After that, 1.5%–33.4% of the data is eliminated. All the sequencing reads were deposited in NCBI Short Read Archive (accession number: SRA065845).

### Correction of reference genome with S0 mutations and mapping raw reads on the new reference genome

To compare S10 and S20 lineages against S0, a new reference genome is needed. When S0 was aligned to MG8, a total of 504 variations were found in S0. These were applied on MG8 to make a new reference genome, MG8_S0. This will be used for calling variations in S10 and S20 lineages. Annotation of the new modified reference genome, MG8_S0, was resolved by using annotation migration tool, Scipio v1.4 [30]. To align Illumina sequencing reads on the MG8 reference genome, BWA (Burrows-Wheeler Aligner) 0.6.1-r104 [31] was used.

### Variant calling and analysis of variants

To find structural variations, SAMtools 0.1.18 [32] was used. Among all the possible variants, we restricted our analysis to the one with at least four sequencing depth and over 0.5 of frequency. To calculate mapping coverage on the reference genome, Bed file format was used. To convert BAM file to Bed, bamToBed in BEDtools package was used with default options. The snpEff package v2.0.5d (http://snpeff.sourceforge.net/) was used to investigate the followings: 1) to identify where SNPs/InDels are located (Intergenic/Exon/Intron); 2) to investigate transitions and transversions; 3) to find synonymous and non-synonymous changes when a codon was changed by SNP.

### Validation of mutations by Sanger sequencing

The loci to be checked by Sanger sequencing were randomly selected from the dataset and subjected to PCR amplification. Successfully amplified loci were treated with ExoSAP-IT® For PCR Product Clean-UP (Affimetrix) and directly capillary-sequenced with primers used in PCR.

## Supporting Information

Figure S1 **Growth rate of the derived strains on oatmeal agar plates.** Growth rate was measured as the colony diameter. At least three replicates were used for each of three independent measurement.
(PDF)

Figure S2 **Germination (black bar) and appressorium formation (white bar) of the derived strains.** At least three replicates were used for each of three independent experiment. Asterisk indicates statistically significant differences, compared to S0.
(PDF)

Figure S3 **Virulence of the derived strains on a susceptible rice cultivar M2O2.** At least three diseased leaves were sampled for evaluation of virulence for each of three independent test.
(PDF)

Figure S4 **DNA fingerprinting of the derived strains and their lesion isolates.** Two different enzymes were used with MGR586 as probe. S20-1 #1 in *EcoR*I panel and S20-2 #2 in *Hind*III panel show degradation of genomic DNA after digestion.
(PDF)

Figure S5 **Length distribution of insertions and deletions (InDel) in the derived strains.** X-axis represents length of InDels (positive value for insertion and negative value for deletion). Y-axis represents the number of InDels having corresponding lengh in X-axis.
(PDF)

Figure S6 **Distribution of mutations across chromosomes among the derived strains.** Each vertical bar represents chromosomes within the derived strain. Horizontal lines within the bar represent the mutations found in the particular position of chromosomes. Green , blue, and red indicate SNP, insertion, and deletion, respectively.
(PDF)

Table S1 **Basic statistics of whole genome sequencing.**
(DOCX)

Table S2 **Average sequencing depth of raw and mapped reads.**
(DOCX)

Table S3 **Percentage of covered nucleotide sites present within the genome of** ***M. oryzae.***
(DOCX)

Table S4 **Proportion of nucleotide sites covered by at least four sequencing reads.**
(DOCX)

Table S5 **List of the mutations that were validated by Sanger sequencing.**
(DOCX)

Table S6 **Distribution of mutations in coding sequences (CDS) and non-coding sequences (nonCDS).**
(DOCX)

Table S7 **Transitions and transversions in the derived strain.**
(DOCX)

Table S8 **List of mutations that are found in coding sequences with T-DNA insertion lines available.**
(DOCX)
