# Supplementary figures and images for: Experimental Evolution Reveals Genome-Wide Spectrum and Dynamics of Mutations in the Rice Blast Fungus, Magnaporthe oryzae
Source: PLoS One. 2013 May 31;8(5):e65416. doi: 10.1371/journal.pone.0065416 (PMC3669265; doi:10.1371/journal.pone.0065416)

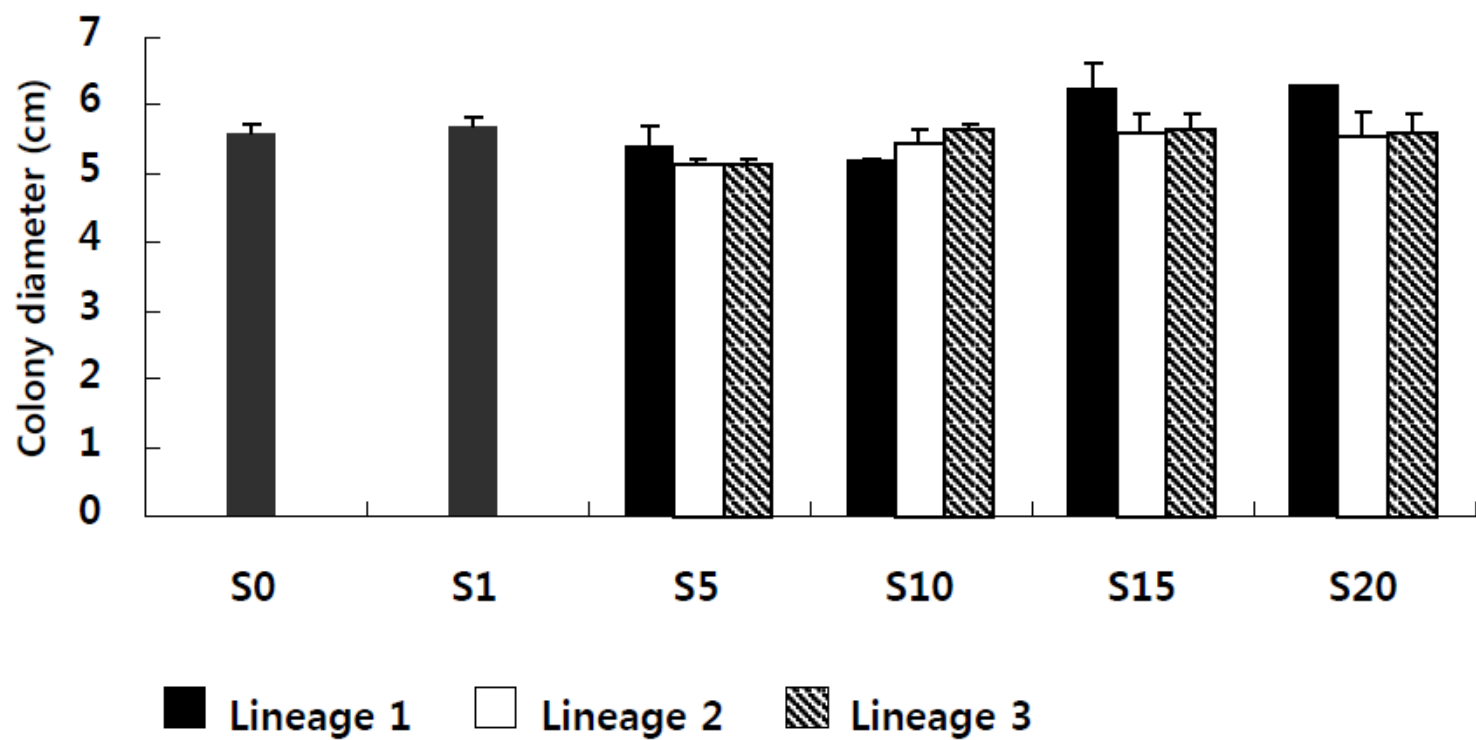

Supplement: Figure S1 — Growth rate of the derived strains on oatmeal agar plates. Growth rate was measured as the colony diameter. At least three replicates were used for each of three independent measurement. (PDF) [file pone.0065416.s001.pdf]

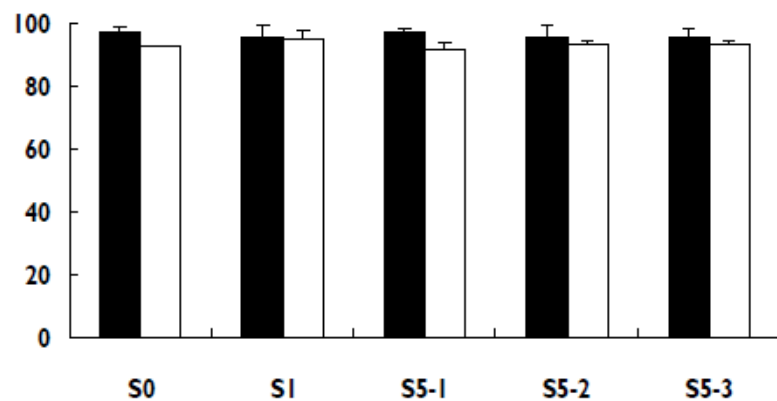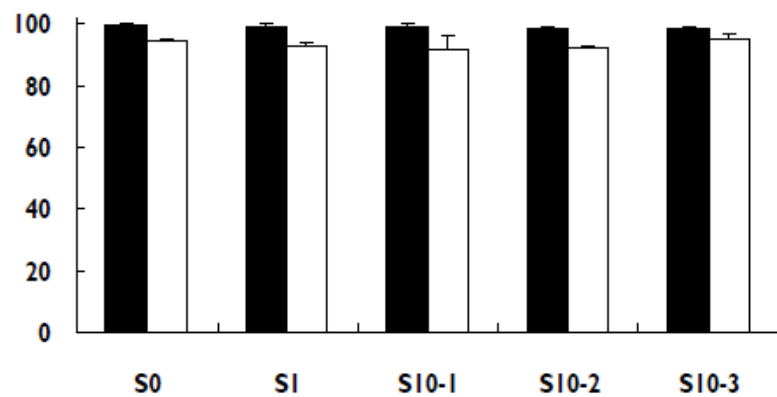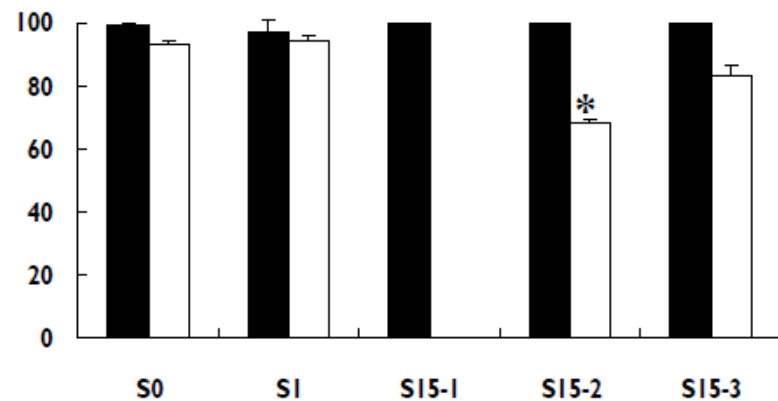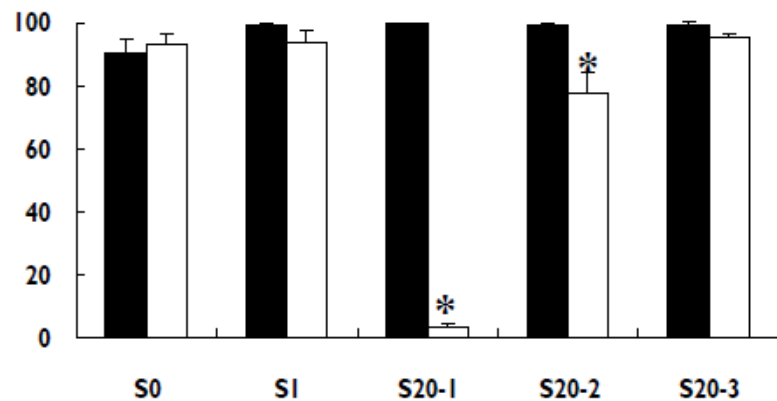

Supplement: Figure S2 — Germination (black bar) and appressorium formation (white bar) of the derived strains. At least three replicates were used for each of three independent experiment. Asterisk indicates statistically significant differences, compared to S0. (PDF) [file pone.0065416.s002.pdf]

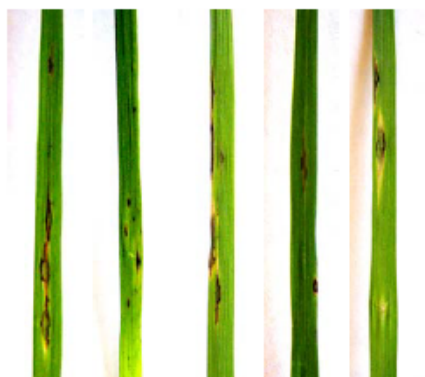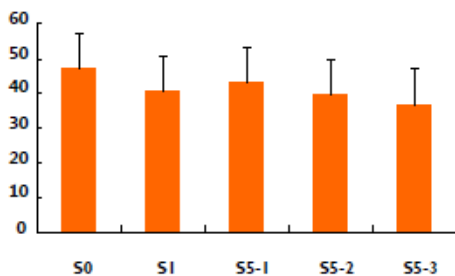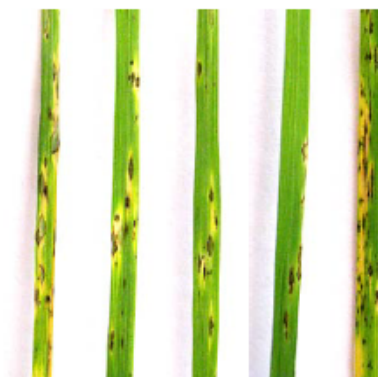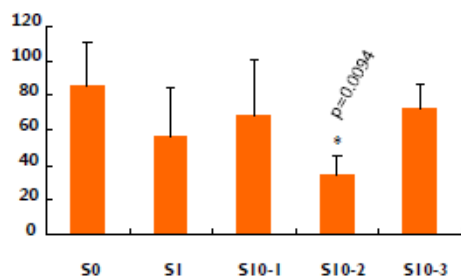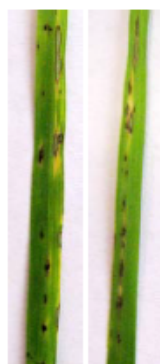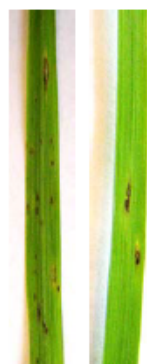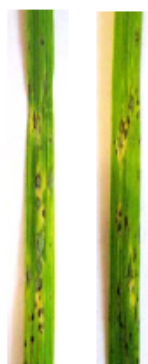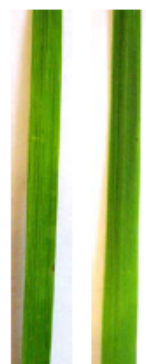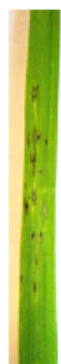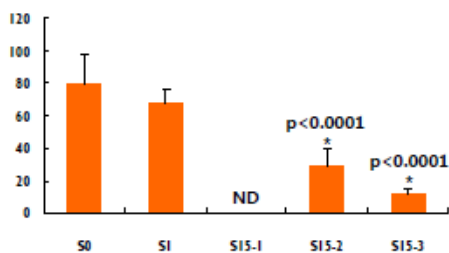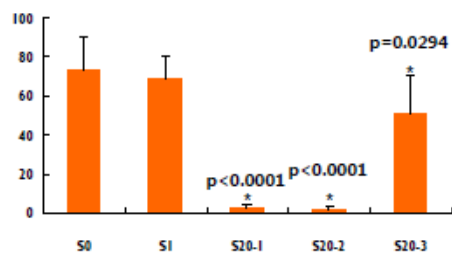

Supplement: Figure S3 — Virulence of the derived strains on a susceptible rice cultivar M2O2. At least three diseased leaves were sampled for evaluation of virulence for each of three independent test. (PDF) [file pone.0065416.s003.pdf]

EcoRI

S0 S1 S1 #1 S20-1 S20-1 #1 S20-2 S20-2 #1 S20-2 #2 S20-3 S20-3 #1 S20-3 #2 S20-3 #3

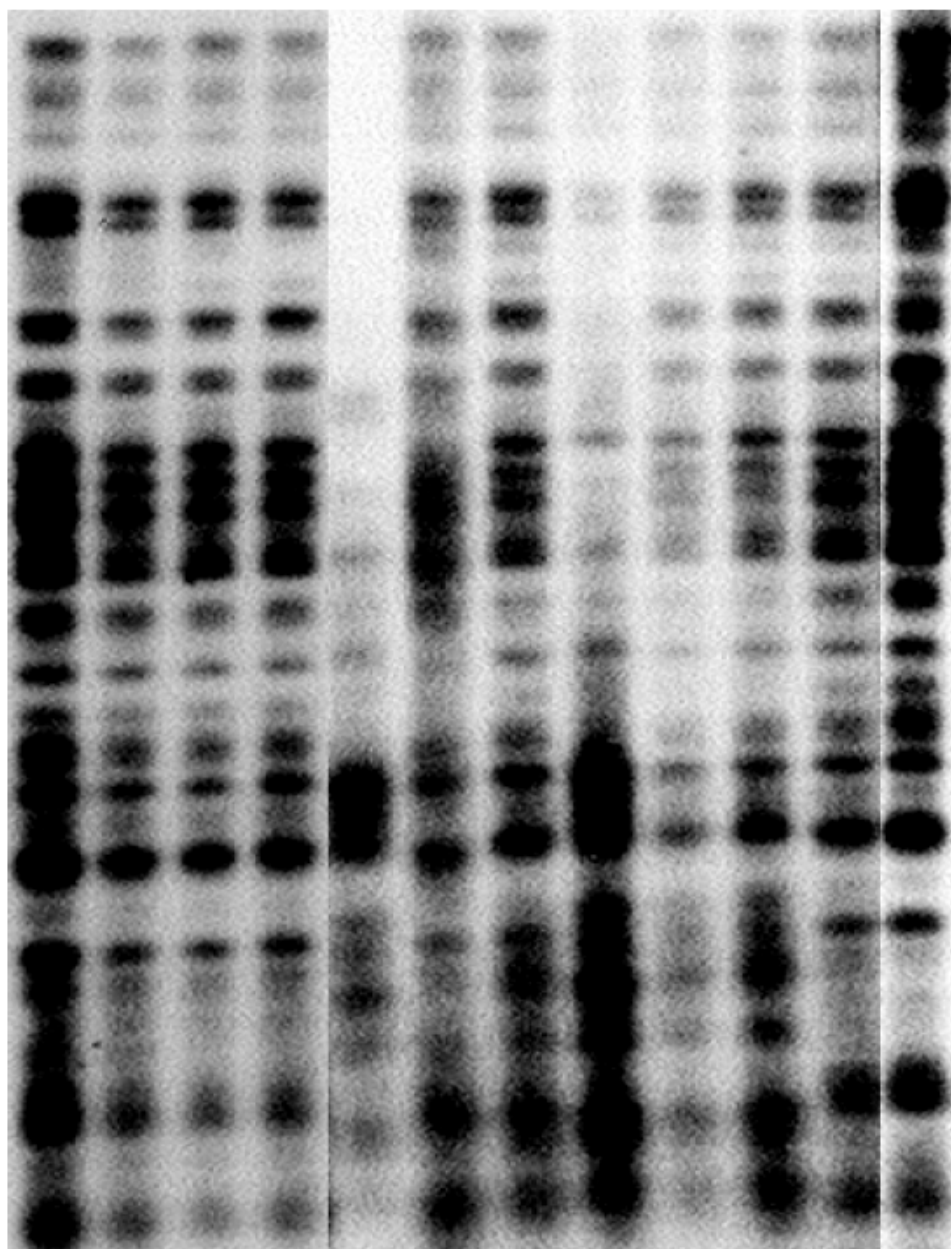

HindIII

S0 S1 S1 #1 S20-1 S20-1 #1 S20-2 S20-2 #1 S20-2 #2 S20-3 S20-3 #1 S20-3 #2 S20-3 #3

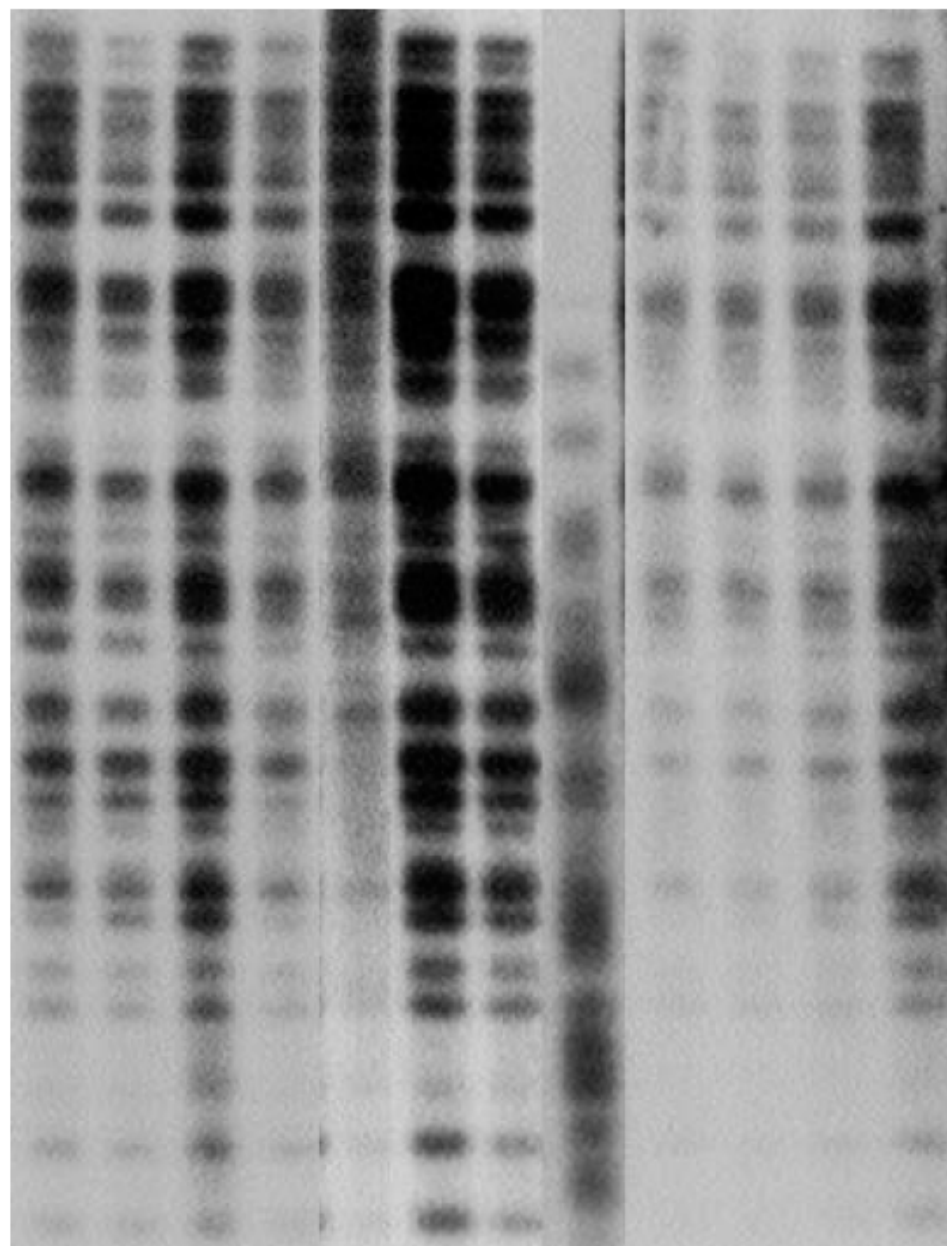

Supplement: Figure S4 — DNA fingerprinting of the derived strains and their lesion isolates. Two different enzymes were used with MGR586 as probe. S20-1 #1 in EcoRI panel and S20-2 #2 in HindIII panel show degradation of genomic DNA after digestion. (PDF) [file pone.0065416.s004.pdf]

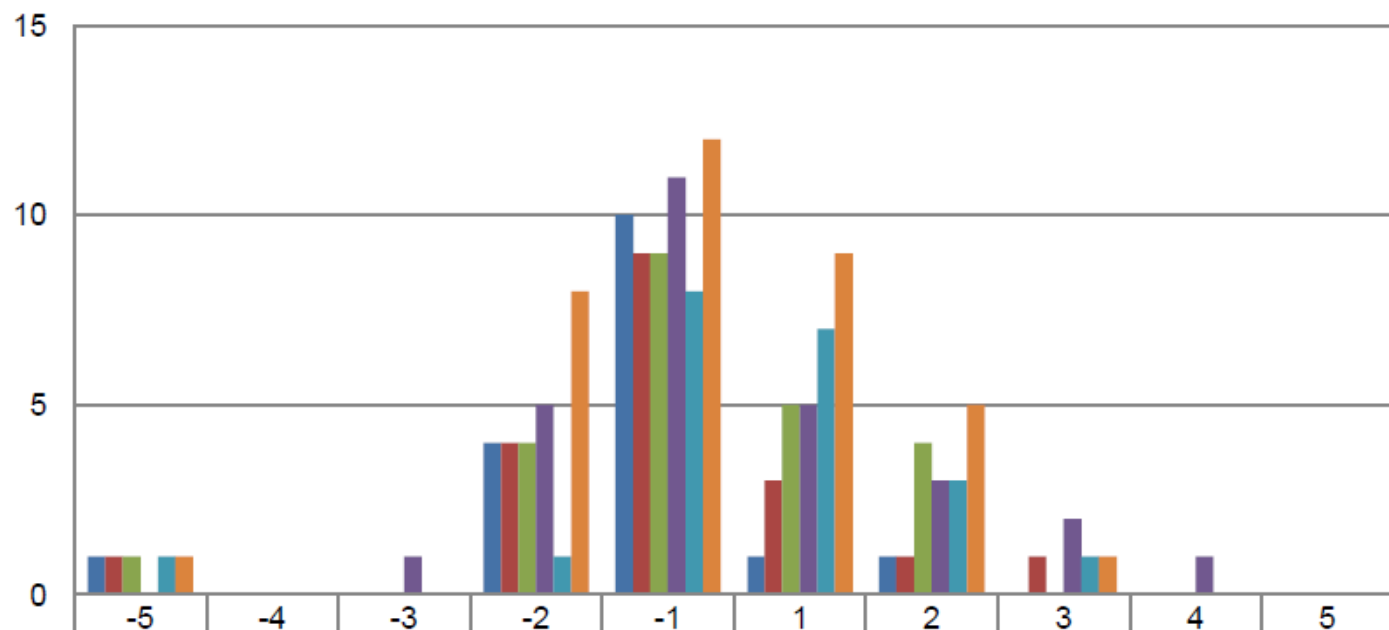

|       | -5 | -4 | -3 | -2 | -1 | 1 | 2 | 3 | 4 | 5 |
|-------|----|----|----|----|----|---|---|---|---|---|
| S10-1 | 1  | 0  | 0  | 4  | 10 | 1 | 1 | 0 | 0 | 0 |
| S10-2 | 1  | 0  | 0  | 4  | 9  | 3 | 1 | 1 | 0 | 0 |
| S10-3 | 1  | 0  | 0  | 4  | 9  | 5 | 4 | 0 | 0 | 0 |
| S20-1 | 0  | 0  | 1  | 5  | 11 | 5 | 3 | 2 | 1 | 0 |
| S20-2 | 1  | 0  | 0  | 1  | 8  | 7 | 3 | 1 | 0 | 0 |
| S20-3 | 1  | 0  | 0  | 8  | 12 | 9 | 5 | 1 | 0 | 0 |

Supplement: Figure S5 — Length distribution of insertions and deletions (InDel) in the derived strains. X-axis represents length of InDels (positive value for insertion and negative value for deletion). Y-axis represents the number of InDels having corresponding lengh in X-axis. (PDF) [file pone.0065416.s005.pdf]

# Strain

S<sub>10-1</sub>

S<sub>10-2</sub>

S<sub>10-3</sub>

S<sub>20-1</sub>

S<sub>20-2</sub>

S<sub>20-3</sub>

1

2

3

4

5

6

7

NA

Chromosome

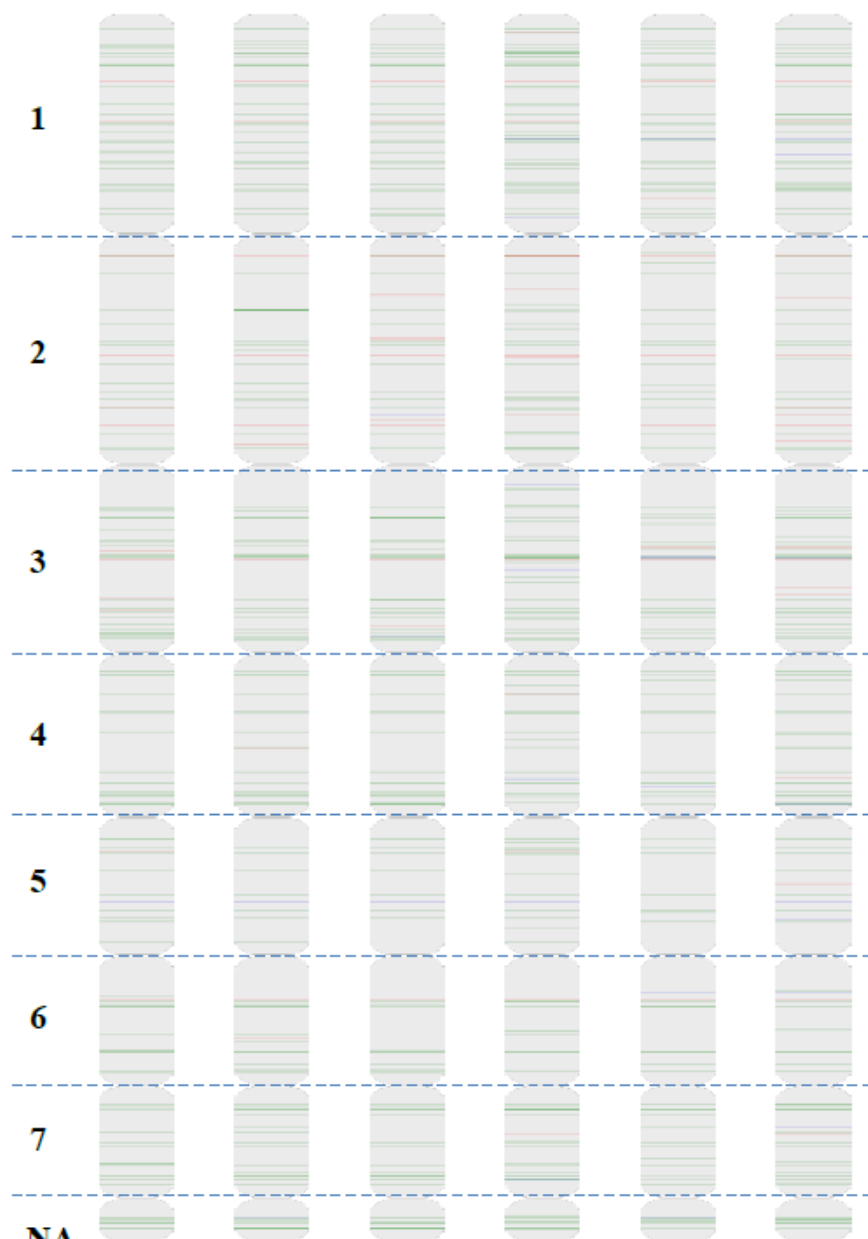

Supplement: Figure S6 — Distribution of mutations across chromosomes among the derived strains. Each vertical bar represents chromosomes within the derived strain. Horizontal lines within the bar represent the mutations found in the particular position of chromosomes. Green , blue, and red indicate SNP, insertion, and deletion, respectively. (PDF) [file pone.0065416.s006.pdf]
