# Supplementary material for: Experimental Evolution Reveals Genome-Wide Spectrum and Dynamics of Mutations in the Rice Blast Fungus, Magnaporthe oryzae
Source: PLoS One. 2013 May 31;8(5):e65416. doi: 10.1371/journal.pone.0065416 (PMC3669265; doi:10.1371/journal.pone.0065416)
Supplement: Table S1 — Basic statistics of whole genome sequencing. (DOCX) [file pone.0065416.s007.docx]

Table S1. Basic statistics of whole genome sequencing

| **Strain** | **Size** | **Raw Read Depth** | **Depth of Aligned Reads** |
| --- | --- | --- | --- |
| S0 | 1295012592 | 31.56 | 29.23 |
| S10-1 | 4095725448 | 99.83 | 82.34 |
| S10-2 | 4156334754 | 101.31 | 76.97 |
| S10-3 | 4090114358 | 99.69 | 75.53 |
| S20-1 | 1706770800 | 41.60 | 37.56 |
| S20-2 | 4095473811 | 99.82 | 76.81 |
| S20-3 | 4048043505 | 98.67 | 80.15 |
