# Supplementary material for: Experimental Evolution Reveals Genome-Wide Spectrum and Dynamics of Mutations in the Rice Blast Fungus, Magnaporthe oryzae
Source: PLoS One. 2013 May 31;8(5):e65416. doi: 10.1371/journal.pone.0065416 (PMC3669265; doi:10.1371/journal.pone.0065416)
Supplement: Table S2 — Average sequencing depth of raw and mapped reads. (DOCX) [file pone.0065416.s008.docx]

Table S2. Average sequencing depth of raw and mapped reads

| **Lineage** | **Size** | **Raw Read Depth** | **Depth of Aligned Reads** |
| --- | --- | --- | --- |
| S0 | 1275597288 | 31.06 | 28.97 |
| S10-1 | 1643712344 | 40.03 | 35.73 |
| S10-2 | 1643712279 | 40.03 | 35.06 |
| S10-3 | 1643712279 | 40.03 | 35.17 |
| S20-1 | 1643712264 | 40.03 | 36.83 |
| S20-2 | 1643712279 | 40.03 | 35.03 |
| S20-3 | 1643712279 | 40.03 | 36.18 |
