# Supplementary material for: Experimental Evolution Reveals Genome-Wide Spectrum and Dynamics of Mutations in the Rice Blast Fungus, Magnaporthe oryzae
Source: PLoS One. 2013 May 31;8(5):e65416. doi: 10.1371/journal.pone.0065416 (PMC3669265; doi:10.1371/journal.pone.0065416)
Supplement: Table S3 — Percentage of covered nucleotide sites present within the genome of M. oryzae. (DOCX) [file pone.0065416.s009.docx]

Table S3. Percentage of covered nucleotide sites present within the genome of *M. oryzae*

| **Lineage** | **Reference  genome size (bp)** | **Covered sites (bp)** | **Ratio** |
| --- | --- | --- | --- |
| S0 | 41062686 | 40958442 | 99.75% |
| S10-1 | 41062692 | 40878231 | 99.55% |
| S10-2 | 41062692 | 40702514 | 99.12% |
| S10-3 | 41062692 | 40569126 | 98.80% |
| S20-1 | 41062692 | 40952069 | 99.73% |
| S20-2 | 41062692 | 39476371 | 96.14% |
| S20-3 | 41062692 | 40501159 | 98.63% |
