# Supplementary material for: Experimental Evolution Reveals Genome-Wide Spectrum and Dynamics of Mutations in the Rice Blast Fungus, Magnaporthe oryzae
Source: PLoS One. 2013 May 31;8(5):e65416. doi: 10.1371/journal.pone.0065416 (PMC3669265; doi:10.1371/journal.pone.0065416)
Supplement: Table S4 — Proportion of nucleotide sites covered by at least four sequencing reads. (DOCX) [file pone.0065416.s010.docx]

Table S4. Proportion of nucleotide sites covered by at least four sequencing reads

| **Lineage** | **Reference  genome size (bp)** | **Over 4x (bp)** | **Ratio** |
| --- | --- | --- | --- |
| S0 | 41062686 | 40906171 | 99.62% |
| S10-1 | 41062690 | 40528236 | 98.70% |
| S10-2 | 41062690 | 39766656 | 96.84% |
| S10-3 | 41062690 | 39316364 | 95.75% |
| S20-1 | 41062690 | 40860128 | 99.51% |
| S20-2 | 41062690 | 36333733 | 88.48% |
| S20-3 | 41062690 | 38988961 | 94.95% |
