# Supplementary material for: Experimental Evolution Reveals Genome-Wide Spectrum and Dynamics of Mutations in the Rice Blast Fungus, Magnaporthe oryzae
Source: PLoS One. 2013 May 31;8(5):e65416. doi: 10.1371/journal.pone.0065416 (PMC3669265; doi:10.1371/journal.pone.0065416)
Supplement: Table S5 — List of the mutations that were validated by Sanger sequencing. (DOCX) [file pone.0065416.s011.docx]

Table S5. List of the mutations that were validated by Sanger sequencing

| **Strain** | **Chromosome** | **Position** | **Change^a^** | | **Frequency** | **Sanger Sequencing^b^** |
| --- | --- | --- | --- | --- | --- | --- |
| S10-1 | supercont8.1 | 811343 | T | A | <1 | Yes |
|  | supercont8.4 | 1774729 | G | A | 1 | Yes |
|  | supercont8.7 | 3158861 | C | T | <1 | Yes |
|  | supercont8.1 | 3158922 | C | T | <1 | Yes |
| S10-2 | supercont8.1 | 811329 | T | A | <1 | Yes |
|  | supercont8.4 | 5213688 | G | A | 1 | Yes |
|  | supercont8.7 | 28760 | T | G | 1 | Yes |
|  | supercont8.6 | 4127585 | A | G | <1 | Yes |
| S10-3 | supercont8.2 | 5876403 | T | C | 1 | Yes |
|  | supercont8.3 | 3001414 | A | G | <1 | No |
| S20-1 | supercont8.6 | 3254939 | A | G | <1 | Yes |
|  | supercont8.6 | 3254962 | T | C | <1 | Yes |
| S20-2 | supercont8.1 | 811617 | G | A | <1 | Yes |
|  | supercont8.1 | 811624 | C | T | <1 | Yes |
|  | supercont8.1 | 811641 | T | G | <1 | Yes |
|  | supercont8.6 | 3256089 | T | C | <1 | Yes |
| S20-3 | Supercont8.1 | 5068128 | T | C | <1 | NA^c^ |
|  | supercont8.6 | 2352336 | T | C | 1 | No |

^a^Change of nucleotide from corrected reference to the evolved genome.

^b^Whether the changes are confirmed by Sanger sequencing.

^c^Not amplificed in PCR.
