# Supplementary material for: Experimental Evolution Reveals Genome-Wide Spectrum and Dynamics of Mutations in the Rice Blast Fungus, Magnaporthe oryzae
Source: PLoS One. 2013 May 31;8(5):e65416. doi: 10.1371/journal.pone.0065416 (PMC3669265; doi:10.1371/journal.pone.0065416)
Supplement: Table S6 — Distribution of mutations in coding sequences (CDS) and non-coding sequences (nonCDS). (DOCX) [file pone.0065416.s012.docx]

Table S6. Distribution of mutations in coding sequences (CDS) and non-coding sequences (nonCDS)

|  | **Type** | **S10-1** | | **S10-2** | | **S10-3** | | **S20-1** | | **S20-2** | | **S20-3** | |
| --- | --- | --- | --- | --- | --- | --- | --- | --- | --- | --- | --- | --- | --- |
|  |  | **CDS** | **nonCDS** | **CDS** | **nonCDS** | **CDS** | **nonCDS** | **CDS** | **nonCDS** | **CDS** | **nonCDS** | **CDS** | **nonCDS** |
| **Observed** | **Insertion** | 4 | 8 | 2 | 13 | 3 | 16 | 5 | 23 | 1 | 15 | 2 | 12 |
|  | **Deletion** | 1 | 13 | 1 | 13 | 1 | 20 | 2 | 22 | 0 | 16 | 2 | 22 |
|  | **SNP** | 66 | 257 | 64 | 237 | 69 | 261 | 83 | 377 | 61 | 248 | 66 | 246 |
| **Expected** | **Insertion** | 4.98 | 7.02 | 6.22 | 8.78 | 7.88 | 11.12 | 11.62 | 16.38 | 6.64 | 9.36 | 5.81 | 8.19 |
|  | **Deletion** | 5.81 | 8.19 | 5.81 | 8.19 | 8.71 | 12.29 | 9.96 | 14.04 | 6.64 | 9.36 | 9.96 | 14.04 |
|  | **SNP** | 134.02 | 188.98 | 124.89 | 176.11 | 136.92 | 193.08 | 190.86 | 269.14 | 128.21 | 180.79 | 129.45 | 182.55 |
