# Supplementary material for: Experimental Evolution Reveals Genome-Wide Spectrum and Dynamics of Mutations in the Rice Blast Fungus, Magnaporthe oryzae
Source: PLoS One. 2013 May 31;8(5):e65416. doi: 10.1371/journal.pone.0065416 (PMC3669265; doi:10.1371/journal.pone.0065416)
Supplement: Table S7 — Transitions and transversions in the derived strain. (DOCX) [file pone.0065416.s013.docx]

Table S7. Transitions and transversions in the derived strain

| **Lineage** | **Transition** | **Transversion** | **Ts/Tv Ratio** |
| --- | --- | --- | --- |
| **S10-1** | 205 | 45 | 4.56 |
| **S10-2** | 193 | 39 | 4.95 |
| **S10-3** | 207 | 43 | 4.81 |
| **S20-1** | 239 | 58 | 4.12 |
| **S20-2** | 158 | 37 | 4.27 |
| **S20-3** | 198 | 41 | 4.83 |
