# Supplementary material for: Experimental Evolution Reveals Genome-Wide Spectrum and Dynamics of Mutations in the Rice Blast Fungus, Magnaporthe oryzae
Source: PLoS One. 2013 May 31;8(5):e65416. doi: 10.1371/journal.pone.0065416 (PMC3669265; doi:10.1371/journal.pone.0065416)
Supplement: Table S8 — List of mutations that are found in coding sequences with T-DNA insertion lines available. (DOCX) [file pone.0065416.s014.docx]

Table S8. List of mutations that are found in coding sequences with T-DNA insertion lines available

| **Locus** | **Strain** | **Chr.** | **Position** | **Change** | | **Type** | **Freq** | **Exon Rank** | **Effect** | **T-DNA** | | | |
| --- | --- | --- | --- | --- | --- | --- | --- | --- | --- | --- | --- | --- | --- |
|  |  |  |  |  |  |  |  |  |  | **ID** | **Position** | **Distance** | **Phenotypic defects** |
| **MGG_01344T0** | **S20-1** | supercont8.2 | 3187723 | A | C | SNP | <1 | 1 | Nonsynonymous | ATMT0207B6 | 5' | 1337bp | Pathogenicity |
| **MGG_03703T0** | **S10-1** | supercont8.4 | 969548 | T | C | SNP | 1 | 2 | Nonsynonymous | ATMT0047B3 | 5' | 306bp | Pathogenicity |
|  | **S10-2** | supercont8.4 | 969548 | T | C | SNP | 1 | 2 | Nonsynonymous |  |  |  |  |
|  | **S10-3** | supercont8.4 | 969548 | T | C | SNP | 1 | 2 | Nonsynonymous |  |  |  |  |
|  | **S20-1** | supercont8.4 | 969548 | T | C | SNP | 1 | 2 | Nonsynonymous |  |  |  |  |
|  | **S20-2** | supercont8.4 | 969548 | T | C | SNP | 1 | 2 | Nonsynonymous |  |  |  |  |
|  | **S20-3** | supercont8.4 | 969548 | T | C | SNP | 1 | 2 | Nonsynonymous |  |  |  |  |
| **MGG_04923T0** | **S20-1** | supercont8.3 | 3965142 | G | A | SNP | 1 | 2 | Nonsynonymous | ATMT0057B2 | 5' | 839bp | Appressorium, Pathogenicity |
| **MGG_06064T0** | **S10-1** | supercont8.3 | 2445604 | G | A | SNP | 1 | 3 | Nonsynonymous | ATMT0045B1 | 5' | 859bp | Appressorium, Pathogenicity |
|  | **S10-2** | supercont8.3 | 2445604 | G | A | SNP | 1 | 3 | Nonsynonymous |  |  |  |  |
|  | **S10-3** | supercont8.3 | 2445604 | G | A | SNP | 1 | 3 | Nonsynonymous |  |  |  |  |
|  | **S20-1** | supercont8.3 | 2445604 | G | A | SNP | 1 | 3 | Nonsynonymous |  |  |  |  |
| **MGG_08208T0** | **S20-1** | supercont8.2 | 6082401 | A | C | SNP | <1 | 1 | Synonymous | ATMT0457B6 | Exon | 0 | No defect |
| **MGG_13324T0** | **S20-1** | supercont8.3 | 67254 | * | +G | INS | 1 | 2 | Frame shift | ATL0379 | Exon | 0 | Not available |
| **MGG_15990T0** | **S20-1** | supercont8.1 | 194398 | * | -ACA | DEL | 1 | 1 | Codon deletion | ATMT0215B6 | 5' | 362bp | Growth, Pathogenicity |
